# Prevalence of mental health disorders and their association with chronic physical diseases in Kuwait

**DOI:** 10.3389/fpsyt.2025.1658457

**Published:** 2025-10-17

**Authors:** Abdullah Al-Ozairi, Mohammad Irshad, Fatmah Alsarraf, Sagarika Raina, Husain Alsaraf, Ebaa Al Ozairi

**Affiliations:** ^1^ Department of Psychiatry, Faculty of Medicine, Kuwait University, Kuwait City, Kuwait; ^2^ Amiri Hospital, Ministry of Health, Kuwait City, Kuwait; ^3^ Dasman Diabetes Institute, Kuwait City, Kuwait; ^4^ Royal College of Surgeons, Dublin, Ireland

**Keywords:** mental disorders, depression, delirium, cancer, hypertension, diabetes

## Abstract

**Objective:**

This study aimed to evaluate the prevalence of mental health disorders and their associations with chronic physical diseases in secondary healthcare settings in Kuwait.

**Methods:**

This retrospective cross-sectional study analyzed data collected from electronic health records of psychiatric care units in secondary healthcare hospitals in Kuwait. Mental health disorders were diagnosed by professionals and documented using the International Classification of Diseases, 10^th^ Revision (ICD-10). We collected both mental and physical health data, along with basic demographic information. Logistic regression models adjusted for age, sex, drug abuse, and developmental disorders were used to examine associations. Results are reported as adjusted odds ratios (AOR) with 95% confidence intervals (CI).

**Results:**

A total of 11921 patient records from psychiatric units in secondary care hospitals were analyzed. Among these patients, 41.1% (n= 4902) had a chronic mental health disorder, with depression being the most common (33.7%, n= 4023). Comorbid chronic mental health disorders and chronic physical diseases were observed in 19.5% (n= 2319) of patients. Patients with chronic physical diseases were 1.8 times more likely to have a chronic mental health disorder compared to those without chronic diseases (AOR=1.8, 95% CI: 1.6–2.0, p< 0.001). Depression was significantly associated with cancer (AOR, 2.9; 95%CI, 2.4–3.6), diabetes (AOR, 2.0; 95%CI, 1.7–2.3), renal disease (AOR, 1.8; 95%CI, 1.5–2.1), hypertension (AOR, 1.7; 95%CI, 1.4–2.0), neurological disease (AOR, 1.6; 95%CI, 1.4–1.8), cardiovascular disease (AOR, 1.5; 95%CI, 1.3–1.8), and respiratory disease (AOR, 1.2; 95%CI, 1.0–1.5). Somatic symptom disorder was significantly associated with neurological disease (AOR, 1.6; 95% CI, 1.3–2.0).

**Conclusions:**

This study revealed a substantial burden of mental health disorders, with depression showing significant associations with multiple chronic physical diseases. However, causal inferences cannot be drawn from this cross-sectional design. These findings are hypothesis-generating and highlight the need for further research on systematic mental health monitoring in secondary care populations.

## 1 Introduction

Mental health disorders contribute substantially to the global burden of morbidity and mortality (1). According to the World Health Organization (WHO), one in every eight people worldwide is living with a mental health disorder (2). In the Middle East, the prevalence of mental illness in the general population ranges from 15.6% to 35.5% (3). In Kuwait, a study reported that 42.7% of patients attending primary health clinics had a mental health disorder (4). Mental health disorders have long been recognized as contributing to poor quality of life, premature mortality, and substantial healthcare costs. Individuals with severe mental health disorders are estimated to die 10 to 20 years earlier than those without such conditions (5). Moreover, patients with both mental and physical health comorbidities are at a higher risk of mortality compared to those with mental health disorders alone (6). The economic burden associated with mental health disorders is immense. In the USA, medical spending to treat adults with mental health disorders reached $106.5 billion in 2019 (7).

Mental health disorders constitute a wide range of conditions, including depression, bipolar disorder, anxiety, psychotic disorders, delirium, and schizophrenia (8). The etiology of mental illness is not yet fully understood but appears to involve a complex interplay of biological, psychological and social factors (9).

Mental health disorders and chronic physical diseases have become two major health challenges, often leading to poorer health outcomes. Numerous studies have reported that people with mental health disorders are at increased risk of developing chronic physical diseases such as cardiovascular disease, cancer, and diabetes (10, 11). For example, depression can cause physiological changes, including stress hormone imbalance, poor blood circulation, and inflammation, all of which may increase the risk of chronic physical disease (12, 13). Conversely, the psychological burden of managing a chronic physical disease can exacerbate mental health problems, creating a cycle of adverse health outcomes (14). Many studies have shown that individuals with diabetes, hypertension, and asthma are more likely to experience psychological distress (15–17).

Psychological support plays a pivotal role in improving outcomes for individuals with chronic physical illnesses and in preventing the onset or progression of these conditions (18). Addressing psychological challenges in patients enhances treatment adherence, promotes healthier lifestyle behaviors, and strengthens self-management capacities, which collectively contribute to improved disease control and overall quality of life (19, 20). However, in clinical care, mostly physicians pay less attention to the interdependence of mental disorders and chronic physical diseases. The primary focus typically remains on managing physical symptoms, ensuring medication adherence, and preventing complications. As a result, mental health issues are often overlooked or underestimated.

In Western countries, mental health status is routinely assessed, and appropriate psychological support is provided (21). Similarly, in Kuwait, mental healthcare services are available within secondary healthcare settings, where professionals assess mental health conditions and provide support to the affected patients (22). However, the prevalence of mental illness in secondary healthcare settings has not yet been reported. Previous local studies, mainly conducted in primary healthcare settings, have been limited to a narrow range of mental health disorders, primarily depression and anxiety (4, 23, 24). Therefore, this study aimed to investigate the broader spectrum of mental health disorders and their associations with chronic physical diseases in secondary healthcare settings in Kuwait.

## 2 Materials and methods

### 2.1 Study design and population

This study is a retrospective cross-sectional analysis of patient data collected from psychiatric care units of secondary healthcare hospitals in Kuwait. Mental health disorders were assessed by professionals and documented in patients’ electronic health records using the International Statistical Classification of Diseases 10^th^ Revision (ICD-10). We extracted data from the electronic medical records of all patients who attended psychiatric care units between January 2016 and December 2019. The extracted data included patient age, sex, mental health diagnoses, physical health conditions, and appointment dates.

The study followed the ethical principles outlined in the Declaration of Helsinki and received approval from the Ethical Committee of the Ministry of Health, Kuwait (No. 1245). To ensure patient confidentiality and data privacy, all identification details were anonymized prior to data extraction for analysis.

### 2.2 Data screening and extraction

Data screening was conducted by two medical graduates, F. Alsarraf and S. Raina, who independently reviewed electronic medical records. Inclusion criteria for the study were individuals aged 18 years or older with complete information on age, sex, and mental and physical health assessment records. We included only the first-visit assessment record for each patient, even if multiple entries were available. Records with missing or invalid entries were excluded to maintain the accuracy and integrity of the analysis.

Mental health disorders were identified from patient files using diagnostic codes based on the 10th revision of the International Statistical Classification of Diseases (ICD-10) (8). These conditions included delirium, psychotic disorder, depression, bipolar disorder, somatic symptom disorder, anxiety disorder, schizophrenia, suicidal behaviors and dementia. All of these are classified within ICD-10^th^ revision as mental disorders. Furthermore, we grouped acute conditions (delirium, and suicidal behaviors) and chronic conditions (depression, schizophrenia, chronic psychosis, anxiety, bipolar disorder, somatic symptom disorder, dementia). We acknowledge that this classification may contribute to higher prevalence estimates and should therefore be interpreted with caution.

We included all defined chronic physical diseases (yes or no), such as cardiovascular disease, respiratory disease, kidney disease, neurological disease, diabetes, hypertension, and cancer.

### 2.3 Statistical analyses

Statistical analyses were conducted using SPSS (IBM, USA, version 29) software. Descriptive statistics were calculated to summarize the demographic and clinical characteristics. Chi-squared tests were then performed between the prevalence of mental health disorders and sociodemographic characteristics or chronic health diseases.

Multivariable logistic regression was used to examine the associations between mental health disorders and chronic physical diseases, controlling for potential confounding variables such as age, sex, developmental disorder, and drug abuse. The results were presented with adjusted odds ratios (AOR) and 95% confidence intervals (CI) calculated. The level of significance was set at p ≤ 0.05 for all statistical tests, and two-tailed p-values were reported.

## 3 Results

**Table 1** presents the distribution of the sociodemographic and clinical characteristics of patients. This study included 11921 patients, with an almost equal gender distribution (51.0% females and 49.0% males). The age range was 18–99 years (mean ± SD: 51.0± 21.3 years).

**Table 1:** Patients demographics, physical and mental health disorders (n= 11921).

	N	%
Age		
18–32 years	3132	26.3
33–50 years	2864	24.0
51–70 years	3081	25.8
>70 years	2844	23.9
Sex		
Male	5845	49.0
Female	6076	51.0
Physical and mental health		
Depression	4023	33.7
Psychosis	1316	11.0
Somatic symptom disorder	686	5.8
Schizophrenia	247	2.1
Anxiety disorder	228	1.9
Bipolar disorder	109	0.9
Delirium	2381	20.0
Suicidal behaviors	1834	15.4
Sleep disorder	1759	14.8
Burn	120	1.0
Drug overdose	1156	9.7
Substance used	1072	9.0
Developmental Disability	31	0.3
Parkinson	80	0.7
Alzheimer	99	0.8
Dementia	148	1.2
Chronic physical diseases	4805	40.3
Neurological disease	1792	15.0
Cardiovascular disease	931	7.8
Respiratory disease	432	3.6
Renal disease	770	6.5
Diabetes	764	6.4
Hypertension	625	5.2
Cancer	435	3.6
Chronic liver disease	54	0.5
Hypothyroidism	75	0.6
Chronic gastrointestinal disease	183	1.5
Epilepsy	259	2.2
Fits	143	1.2
Seizures	94	0.8
Orthopedics	114	1.0
Injuries-RTA	225	1.9
Injured-other causes	550	4.6
Assault	23	0.2

### 3.1 Mental health disorders

Of the total 11921 patients, 64.9% (n= 7735) had at least one mental health disorder when all ICD-10 mental health conditions were grouped (including both chronic disorders and acute or transient presentations such as delirium and suicidal behaviors). When limited to chronic mental disorders only (e.g., depressive disorders, schizophrenia, bipolar disorder, anxiety disorders, somatic symptom disorder, chronic psychosis), the prevalence was 41.1% (n = 4,902). Acute or transient mental health conditions (e.g., delirium, suicidal behaviors) accounted for 23.8% (n = 2,833), whereas 35.1% (n = 4,183) of the total sample had no recorded mental health disorder.

Among these with chronic mental disorders, 19.7% had one disorder, 13.3% had two disorders, and 8.2% had more than two disorders. The most common disorder was depression (33.7%). Other disorders were psychosis (11.0%), somatic symptom disorder (5.8%), schizophrenia (2.1%), anxiety (1.9%), and bipolar disorder (0.9%). Among acute mental disorders, the most common were delirium (20.0%) followed by suicidal behaviors (15.4%). Neurological conditions, including epilepsy (2.2%), dementia (1.2%), and seizures (0.8%), were also observed.

Chronic mental health disorders were more prevalent among females than males (45.2% vs 36.9%), whereas acute mental health disorders were slightly more prevalence in males compared to females (26.7% vs 20.9%) (χ2 = 97.9; p= 0.001). Across age groups, chronic disorders were most frequent among those aged 51–70 years (46.0%) and 33–50 years (41.7%), while acute disorders were most prominent among those aged >70 years (32.9%) (χ² = 356.1, p <.001) (**Supplementary Table 1**).

### 3.2 Mental health disorders and chronic physical diseases

In this cohort, the overall prevalence of chronic physical disease was 40.3%. These included neurological disease (15.0%), cardiovascular disease (7.8%), respiratory disease (3.6%), renal disease (6.5%), diabetes (6.4%), hypertension (5.2%), and cancer (3.6%). A small proportion of patients had other chronic conditions such as liver disease (0.5%), gastrointestinal disease (1.5%), and hypothyroidism (0.6%). Of the total patients, 19.5% (n= 2319) had comorbid chronic mental health disorders and chronic physical diseases, whereas 9.6% (n=1143) had comorbid chronic physical diseases and acute mental health disorders. In adjusted regression analyses, the presence of chronic physical disease was associated with a 1.8-fold higher likelihood of chronic mental health disorders (AOR=1.8, 95% CI: 1.6–2.0, p < 0.001), but showed a week association with acute mental health disorders (AOR=1.1, 95% CI: 1.0–1.2, p=0.045).

### 3.3 Sensitivity and specificity between chronic physical diseases and mental health disorders

Chronic physical disease moderately identified chronic mental disorders, with a sensitivity of 47.3% (95% CI: 46.0–48.7) and specificity of 64.6% (95% CI: 64.0–65.2), but its ability to identify acute mental disorders was lower, with a sensitivity of 40.3% (95% CI: 38.6–42.0) and specificity of 59.7% (95% CI: 58.7–60.7). When using mental disorders to predict chronic physical disease, chronic mental disorders had similar sensitivity (48.3%, 95% CI: 46.9–49.7) but slightly lower specificity (63.7%, 95% CI: 62.6–64.8), whereas acute mental disorders had low sensitivity (23.8%, 95% CI: 22.7–25.0) and higher specificity (76.3%, 95% CI: 75.3–77.2). These results indicate that chronic physical disease is a modest predictor of chronic mental disorders and less effective for acute mental disorders, while acute mental disorders are poor predictors of chronic physical disease.

### 3.4 Disease-specific prevalence and associations

**Figure 1:**
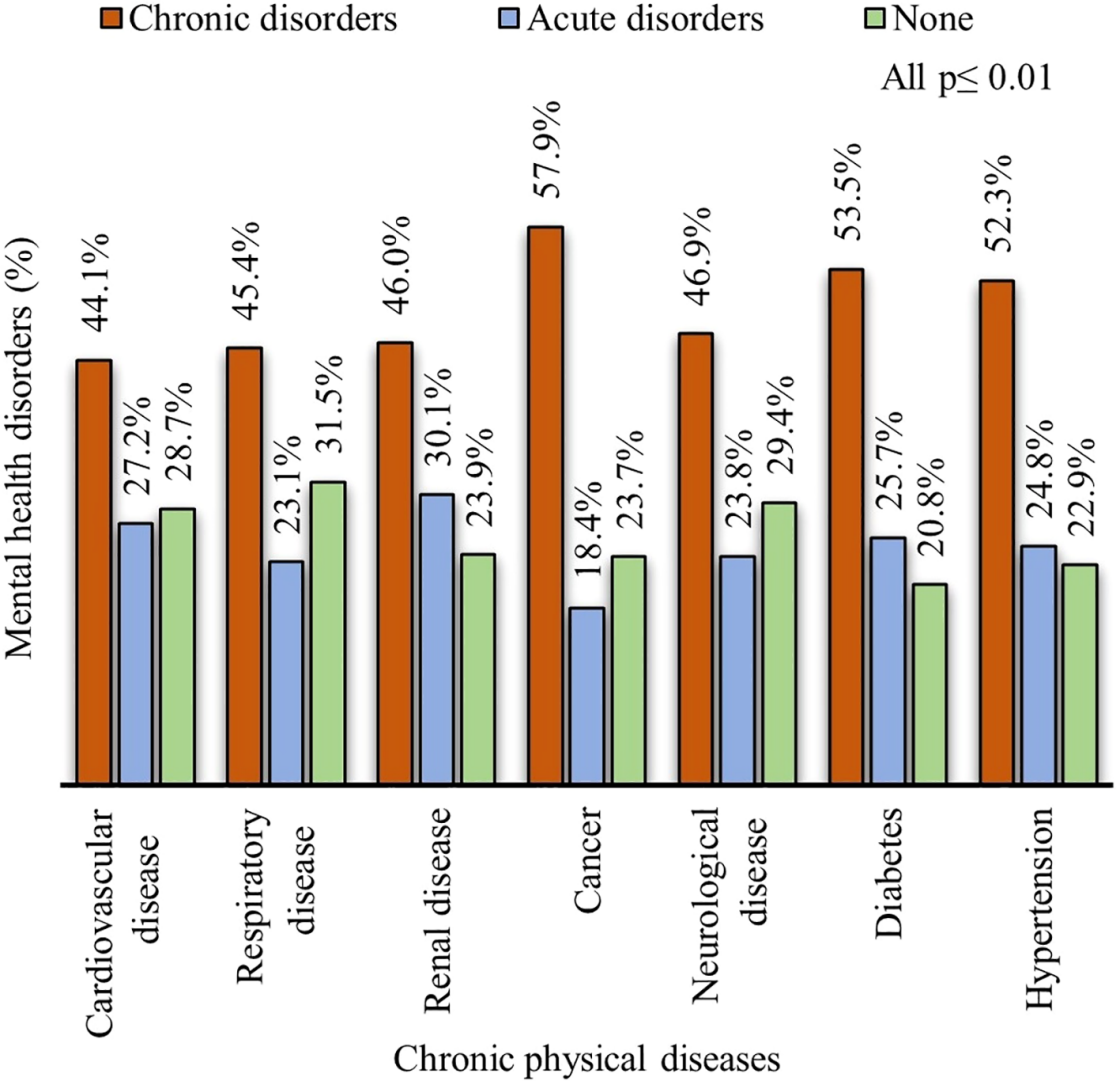
Prevalence of mental health disorders among patients with chronic physical diseases.

Disease-specific analyses showed higher prevalence of chronic mental health disorders among patients with diabetes (53.5%), hypertension (52.3%), cancer (57.9%), renal disease (46.0%), cardiovascular disease (44.1%), neurological disease (46.9%), and respiratory disease (45.4%) (**Figure 1**).

**Table 2** presents the most common mental health disorders among patients with chronic physical diseases and their associations. Depression was the most common disorder among patients with cancer (53.8%), diabetes (45.4%), renal disease (41.8%), hypertension (41.0%), neurological disease (39.5%), cardiovascular disease (37.2%), and respiratory disease (33.1%). In contrast, psychosis (9.8–12.7%), somatic symptom disorder (2.9–7.5%), anxiety (1.1–2.7%), and bipolar disorder (0.2–1.4%) were comparatively less prevalent across chronic physical diseases. Delirium was also common among patients with chronic physical diseases (14.3 – 24.9%). Some patients with delirium were also coded under chronic psychiatric disorders (approximately 14.5%), reflecting overlap in electronic health record coding.

**Table 2:** Association between common mental disorders and chronic health diseases.

	Comorbid	Model 1		Model 2	
Depression	n (%)	OR (95% CI)	p-value	AOR (95% CI)	p-value
Cardiovascular disease	346 (37.2)	1.4 (1.2, 1.6)	<0.001	1.5 (1.3, 1.8)	<0.001
Respiratory disease	143 (33.1)	1.2 (1.0, 1.4)	0.135	1.2 (1.0, 1.5)	0.047
Renal disease	322 (41.8)	1.7 (1.5, 2.0)	<0.001	1.8 (1.5, 2.1)	<0.001
Neurological disease	708 (39.5)	1.5 (1.4, 1.7)	<0.001	1.6 (1.4, 1.8)	<0.001
Cancer	234 (53.8)	2.8 (2.3, 3.3)	<0.001	2.9 (2.4, 3.6)	<0.001
Diabetes	347 (45.4)	2.0 (1.7, 2.3)	<0.001	2.0 (1.7, 2.3)	<0.001
Hypertension	256 (41.0)	1.6 (1.4, 1.9)	<0.001	1.7 (1.4, 2.0)	<0.001
Psychosis	n (%)	OR (95% CI)	p-value	AOR (95% CI)	p-value
Cardiovascular disease	102 (11.0)	1.0 (0.8, 1.2)	0.844	0.8 (0.6, 1.0)	0.159
Respiratory disease	48 (11.1)	1.0 (0.7, 1.4)	0.969	0.9 (0.6, 1.2)	0.363
Renal disease	98 (12.7)	1.2 (0.9, 1.5)	0.196	1.0 (0.8, 1.2)	0.790
Neurological disease	203 (11.3)	1.0 (0.9, 1.2)	0.851	0.9 (0.8, 1.1)	0.359
Cancer	50 (11.5)	1.0 (0.8, 1.4)	0.836	0.9 (0.6, 1.2)	0.423
Diabetes	75 (9.8)	0.9 (0.7, 1.1)	0.256	0.8 (0.6, 1.0)	0.130
Hypertension	69 (11.0)	1.0 (0.8, 1.3)	0.920	0.8 (0.6, 1.1)	0.161
Somatic symptom disorder	n (%)	OR (95% CI)	p-value	AOR (95% CI)	p-value
Cardiovascular disease	49 (5.3)	0.9 (0.7, 1.3)	0.731	1.3 (1.0, 1.8)	0.094
Respiratory disease	22 (5.1)	0.9 (0.6, 1.4)	0.695	1.1 (0.7, 1.8)	0.574
Renal disease	22 (2.9)	0.5 (0.3, 0.8)	0.002	0.7 (0.4, 1.0)	0.069
Neurological disease	134 (7.5)	1.4 (1.1, 1.7)	0.002	1.6 (1.3, 2.0)	<0.001
Cancer	18 (4.1)	0.7 (0.5, 1.2)	0.214	0.9 (0.6, 1.5)	0.669
Diabetes	36 (4.7)	0.8 (0.6, 1.2)	0.341	1.0 (0.7, 1.4)	0.949
Hypertension	35 (5.6)	1.0 (0.7, 1.4)	0.947	1.3 (0.9, 1.9)	0.131
Delirium	n (%)	OR (95% CI)	p-value	AOR (95% CI)	p-value
Cardiovascular disease	212 (22.8)	1.2 (1.0, 1.4)	0.038	0.8 (0.7, 0.9)	0.010
Respiratory disease	85 (19.7)	1.0 (0.8, 1.3)	0.922	0.7 (0.7, 1.0)	0.021
Renal disease	192 (24.9)	1.3 (1.1, 1.6)	0.001	0.9 (0.8, 1.1)	0.393
Neurological disease	360 (20.1)	1.0 (0.9, 1.1)	0.916	0.9 (0.8, 1.0)	0.111
Cancer	62 (14.3)	0.7 (0.5, 0.9)	0.004	0.5 (0.4, 0.7)	<0.001
Diabetes	168 (22.0)	1.1 (0.9, 1.4)	0.165	0.9 (0.8, 1.1)	0.387
Hypertension	129 (20.6)	1.0 (0.9, 1.3)	0.644	0.7 (0.6, 0.9)	0.005

Model 1: Unadjusted; Model 2: Adjusted for age, sex, developmental disorder, and drug abuse.

OR, Odds Ratio; AOR, Adjusted Odds Ratio; 95% CI, 95% Confidence Interval.

Reference group: Patients without any chronic disease.

In adjusted regression analysis, depression was strongly associated with cancer (AOR, 2.9; 95% CI, 2.4–3.6; p< 0.001), diabetes (AOR, 2.0; 95% CI, 1.7–2.3; p< 0.001), renal disease (AOR, 1.8; 95% CI, 1.5–2.1; p< 0.001), hypertension (AOR, 1.7; 95% CI, 1.4–2.0; p< 0.001), neurological disease (AOR, 1.6; 95% CI, 1.4–1.8; p< 0.001) and cardiovascular disease (AOR, 1.5; 95%CI, 1.3–1.8; p< 0.001). Somatic symptom disorder was associated with neurological disease (AOR, 1.6; 95% CI, 1.3–2.0; p< 0.003). Psychosis showed no significant associations with chronic physical diseases in this cohort. Delirium was inversely associated with cardiovascular disease (AOR=0.8, 95% CI: 0.7–0.9, p = 0.01), respiratory disease (AOR=0.7, 95% CI: 0.7–1.0, p = 0.021), cancer (AOR=0.5, 95% CI: 0.4–0.7, p < 0.001), and hypertension (AOR=0.7, 95% CI: 0.6–0.9, p = 0.005).

## 4 Discussion

We explored all types of mental health disorders and chronic physical diseases in a large cohort dataset from secondary care hospitals in Kuwait. Our study contributes by providing a large dataset with clinician diagnoses, highlighting comorbidity patterns with chronic diseases. In this cohort of patients attending psychiatric units in secondary care hospitals in Kuwait, 64.9% had at least one mental health diagnosis when all ICD-10 mental health conditions were considered, while 41.2% had a chronic psychiatric disorder. Approximately 40.3% of the cohort had a chronic physical disease.

The prevalence of chronic mental health disorders in our study was slightly lower than that reported in a recent Kuwait primary-care study (42.7%) (4), but aligns with estimates from other secondary care hospital settings, where chronic psychiatric disorders typically account for 40–45% of patients (25).

For broader context, hospital-based studies internationally have reported comparable ranges: for example, a Puerto Rican hospital system integrating clinical health psychology services found 53% of inpatients had a mental disorder (26), while systematic reviews show prevalence across general practice populations varying widely (2.4–56.3%) depending on setting and diagnostic methodology (27). These comparisons should be interpreted cautiously because differences in care level (primary vs. secondary), referral patterns, and coding practices can substantially influence recorded prevalence.

In this study, most patients had depression, while anxiety was relatively less common. The prevalence of depression observed in this study was slightly higher than that reported in primary health clinics in Kuwait (4). This difference may be explained by the secondary healthcare setting of our study, which typically manages patients with more complex conditions compared to primary healthcare clinics. The prevalence of depression in our study was comparatively higher than developed countries (24.0%) but lower than developing countries (38.0%) in outpatient clinical settings (28). We also observed acute disorders, with delirium (20.0%) being common. The prevalence of delirium was almost equal among patients with chronic physical diseases (20.1%) and those without chronic physical diseases (19.9%). For comparison, international hospital studies have generally reported delirium prevalence ranging from 9% to 32% in general acute care settings (29, 30). Our estimates is lower than that reported in meta-analyses of critically ill patients (82%) (31), and older cancer patients (61.4%) (32). Another meta-analysis in patients with diabetes found delirium prevalence of 31% in prospective cohort studies and 26% in retrospective studies (33). Overall, our study highlights the prevalence of delirium in Kuwait, which should not be overlooked in clinical practice. As reported, delirium is a severe neuropsychiatric syndrome arising from disruptions in neuroinflammation, vascular function, metabolism, and neurotransmission, leading to acute impairments in attention, awareness, and cognition (34).

Conversely, we found a relatively low prevalence of anxiety disorder compared to international and regional studies (35, 36). This may be due to anxiety symptoms often recorded under other diagnostic categories or overshadowed by more severe disorders or overlap with somatic presentations (37). Additional contributing factors may include under-diagnosis, coding bias, and under-recording in EHR workflows. These issues highlight the need for systematic diagnostic and EHR coding practices that minimize overlap with other disorders and improve the accuracy of prevalence estimates.

This study also highlights the sex- and age-related patterns in mental health disorder. Chronic mental health disorders were more common among women than men, aligning with established evidence that women are more vulnerable, possibly due to social and cultural factors (38). Across age groups, the 51–70-year group showed the highest prevalence of chronic disorders compared to younger and older age groups, plausibly reflecting cumulative exposure to risk factors and comorbidities over the life course.

Of the total sample, 19.5% of patients had both chronic mental health disorders and chronic health diseases. The prevalence of comorbid mental illness and chronic physical diseases in our study was lower than that reported in a meta-analysis from Africa (45.8%), Asia (37.0%) and the USA (26.8%) (39). However, regression analyses showed that the presence of chronic physical disease was associated with a 1.8-fold higher likelihood of chronic mental health disorders. Depression alone was more common among patients with chronic diseases (39.7%), compared to those without chronic disease (29.7%), with significantly higher odds among patients with hypertension, renal disease, diabetes, and cancer. The comorbidity of depression was 41.0% in patients with hypertension and 45.4% in patients with diabetes. In comparison, a recent local study conducted in a public primary healthcare setting reported higher rates of comorbid depression with hypertension (54%) and type 2 diabetes (81%) (40). Similarly, a study from Pakistan reported higher prevalence of depression among cancer patients (67%), but lower prevalence in patients with diabetes (38%) and cardiovascular diseases (33%), than our study (41). Many studies have identified depression as an important risk factor associated with chronic physical diseases, including cardiovascular disease (42), diabetes (17), hypertension (43), and kidney disease (44). People with diabetes and cardiovascular disease consistently exhibited higher rates of depression (45, 46). Conversely, a retrospective cohort study suggested that depression may also increase the risk of cancer (47). Likewise, the relationship between depression and neurological diseases is well-documented (48).

In this study, somatic symptom disorders were comparatively uncommon in both patients with and without chronic physical diseases. This finding align with a previous study reporting 6.8% prevalence of somatic symptom disorder among patients with major medical disorders (49). Despite its lower prevalence, somatic symptom disorder was significantly associated with neurological diseases, suggesting that brain tissue damage, such as that seen in stroke, may contribute to somatic symptom disorder (50). Similarly, psychosis was observed in approximately 11% of patients, almost equally distributed among patients with and without chronic physical diseases. In contrast, a recent study from the UK reported a lower prevalence of psychosis (3%) in primary care mental health services (51), it may be higher in healthcare settings due to the presence of comorbid mental disorders (52).

Overall, the high prevalence of mental health disorders in this cohort underscores the substantial mental health burden in clinical populations. Nearly one-fifth of patients had comorbid chronic physical diseases, which were associated with an increased likelihood of mental health disorders. Depression showed particularly strong associations with chronic physical conditions, highlighting the need of systematic mental health screening among these patients. Delirium was also prevalent in patients, highlighting the need of careful monitoring of acute cognitive disturbances in clinical practice.

This study also has limitations. First, the data were collected retrospectively, which lacked information on patients’ socioeconomic status and severity of psychological symptoms, both of which may affect associations with chronic physical diseases. Second, information on treatment approaches for mental illness before visits to secondary care hospitals was also unavailable, limiting our ability to assess non-pharmacological interventions. Third, because data were drawn from psychiatric units in secondary hospitals, referral bias is likely, with more severe or complex patients overrepresented compared to community or primary care settings; this limits the generalizability of our findings. Fourth, delirium is an acute, transient syndrome that may have been coded alongside chronic psychiatric diagnoses in our data set. This coding overlap can inflate prevalence estimates and limits direct comparability with studies using strictly separated diagnostic categories; therefore, delirium prevalence should be interpreted cautiously. Finally, the cross-sectional design limits causal inference.

In conclusion, this study reveals a substantial burden of mental health disorders, especially depression, among patients attending secondary healthcare psychiatric units in Kuwait. Strong associations were observed between depression and several chronic physical diseases, but causal relationships cannot be inferred from this cross-sectional design. These findings are hypothesis-generating and suggest that routine monitoring of mental health among patients with chronic diseases warrants further evaluation. However, given the limitations of retrospective electronic health record data and potential referral and documentation biases, these results should be interpreted cautiously, and future prospective studies are needed to confirm these associations. Future work should validate routine ICD-10 coding practices in psychiatric units and explore how referral criteria and coding conventions influence prevalence estimates.

## Data Availability

The original contributions presented in the study are included in the article/**Supplementary Material**. Further inquiries can be directed to the corresponding authors.
