# Supplementary material for: Prevalence of mental health disorders and their association with chronic physical diseases in Kuwait
Source: Front Psychiatry. 2025 Oct 17;16:1658457. doi: 10.3389/fpsyt.2025.1658457 (PMC12593469; doi:10.3389/fpsyt.2025.1658457)
Supplement: Supplementary file 1 [file Table1.docx]

Supplementary Table 1: Prevalence of mental health disorders stratified by sex and age.

|  | Mental health disorders | | |  |  |
| --- | --- | --- | --- | --- | --- |
|  | CHRONIC | ACUTE | NONE |  |  |
|  | n (%) | n (%) | n (%) | ꭓ2 | p-value |
| Overall | 4902 (41.1) | 2833 (23.8) | 4186 (35.1) | - | - |
| Sex |  |  |  |  |  |
| Male | 2156 (36.9) | 1563 (26.7) | 2126 (36.4) | 97.9 | <0.001 |
| Female | 2746 (45.2) | 1270 (20.9) | 2060 (33.9) |  |  |
| % within Sex |  |  |  |  |  |
| Age groups |  |  |  |  |  |
| 18-32 Years | 1238 (39.5) | 518 (16.5) | 1376 (43.9) | 356.1 | <0.001 |
| 33- 50 Years | 1193 (41.7) | 586 (20.5) | 1085 (37.9) |  |  |
| 51- 70 Years | 1416 (46.0) | 792 (25.7) | 873 (28.3) |  |  |
| >70 Years | 1055 (37.1) | 937 (32.9) | 852 (30.0) |  |  |
| % within Age groups |  |  |  |  |  |
